# An explainable hybrid framework for early detection of cardiovascular diseases using Categorical Boosting and Bees algorithm

**DOI:** 10.1038/s41598-025-28514-4

**Published:** 2025-12-13

**Authors:** Jayanta Sen, Sweta Bhattacharya

**Affiliations:** School of Computer Science Engineering and Information Systems (SCORE), Vellore Institute of Technology, Vellore, Tamil Nadu 632014 India

**Keywords:** Cardiovascular disease, Machine learning, CatBoost, BEES, Explainable AI, Computational science, Computer science, Information technology, Engineering, Cardiology, Cardiovascular biology, Cardiovascular diseases, Heart failure

## Abstract

Cardiovascular disease (CVD) remains one of the leading causes of death worldwide, claiming millions of lives each year. The early detection of CVD enables healthcare professionals to make informed decisions about the patient’s health. Machine learning (ML)- based frameworks have been extremely popular in predicting diseases. However, results generated from traditional ML models are “black-box,” lacking transparency and interpretability. The objective of the present study is to develop an ML framework that detects CVD with promising accuracy and, further, provide interpretability to the generated outcomes to ensure targeted therapies. The Framingham, Massachusetts CVD dataset, which is publicly available from the Kaggle Repository, is used in this study. As part of the data pre-processing, the Random Oversampling (RO) technique is applied to overcome the data imbalance problem, followed by Pearson Correlation analysis to understand the correlation between attributes. Then, the Min–Max scaling technique is used for data normalization. The pre-processed data is fed into a hybrid ML framework incorporating the Categorical Boosting (CatBoost) and BEEs algorithms to achieve optimized CVD prediction results. The proposed Hybrid model yielded 98.04% accuracy, a Precision of 97.09%, a Recall of 98.96%, an F1-score of 98.02%, and a Specificity of 97.16%, with a total execution time of 26.6580 s. The proposed model outperformed contemporary state-of-the-art algorithms, considering most evaluation metrics. Additionally, Explainable Artificial Intelligence (XAI) techniques, such as LIME and SHAP, are implemented to identify the contribution of the most significant attributes towards the occurrence of CVD, offering valuable insights into the detection of the disease and enabling healthcare providers to make accurate and timely treatment decisions.

## Introduction

Cardiovascular disease (CVD) is a condition caused by blood channel obstruction that causes chest pain and heart attacks, leading to major heart-related diseases like heart failure, which can also have fatal consequences^1^. With 15 million deaths recorded in 2015, CVD has continuously been the leading cause of mortality for the previous 15 years^2^. A study from January 2017 confirmed that CVD is the primary cause of global death. By 2020, the World Health Organization estimated that CVD would account for approximately 17.9 million deaths annually worldwide^3^. The death toll from coronary heart disease has been steadily rising, with projections indicating that global deaths from CVD could exceed 23.6 million by 2030, solidifying its position as the most significant cause of mortality worldwide. Cerebrovascular disease, peripheral arterial disease, coronary heart disease, transient ischemic attacks, pulmonary embolism, vascular illnesses, congenital heart defects, chronic heart conditions, deep vein thrombosis, and strokes are some of the prevalent cardiovascular diseases^4^. Diagnosing cardiac disease is further complicated by various risk variables, including irregular pulse rates, high blood pressure, abnormal cholesterol levels, and hypertension. Thus, early detection of cardiovascular diseases (CVD) is crucial for mitigating such an impact. Machine learning (ML), a rapidly advancing field within Artificial Intelligence (AI), has proven valuable in analyzing large datasets from various domains, including healthcare^5^. Hybrid deep learning models that utilise adaptive dropout and optimisation techniques have demonstrated improved convergence and classification accuracy in EEG-based emotion recognition^6^. Consequently, several models are amalgamated and used for precise and prompt prediction. The hybrid models demonstrate a superior capacity for predicting cardiac disease relative to traditional methods^7^. Data mining is one of the methods that significantly improves disease detection and diagnosis. Early identification of CVD not only saves lives but also reduces healthcare costs. Data analysis techniques may carry out this activity successfully and economically by employing classification algorithms, a crucial part of medical research. In this study, we built a predictive model using the Categorical Boosting classifier (CatBoost) and the Bees optimization Algorithm hybrid model through binary classification and supervised learning of patient CVD data. The Categorical Boosting algorithm offers various benefits, especially in its ability to handle categorical variables, eliminating manual processing requirements. It involves target encoding to convert the categorical features into numerical values. Also, overfitting issues are prevented, yielding enhanced accuracy. The integration of the Bees algorithm further improves the model due to its ability for multimodal functional optimization, in the process of finding the global optima within the multiple possible local optima. The algorithm can also be scaled as per the complexity level of the problem in order to arrive at the best possible approach. We approached the problem by splitting the dataset into training and testing sets, after data preparation and preprocessing, applying it to a hybrid model, and assessing its accuracy. Explainable Artificial Intelligence (XAI) contains ways to make AI model predictions clear to humans, which solves the concerns with regular “black-box” models. XAI increases trust and accountability by making things clearer and simpler to grasp, which enables professionals to examine predictions before acting on them. Two major XAI approaches are Shapley Additive Explanations (SHAP) and Local Interpretable Model-agnostic Explanations (LIME). SHAP shows how each feature changes a prediction, whereas LIME shows how a model works in a way that is easy to understand. SHAP and LIME make it simpler to grasp the model by delivering explanations at both the global and local feature levels. This makes it easier to find things in real time. These explanations help clinicians quickly grasp how the model’s findings match up with what they already know about medicine. This helps them make quick and correct treatment choices and fosters trust and accountability. This process allowed us to create a highly accurate pattern for predicting individuals’ CVD risks. By following this approach, hostile risk factors for CVD can be identified precisely. This research also aimed to predict the risks and causes of CVD across generations. The integration of informatics in healthcare, particularly for Cardiovascular disease. Ultimately, this leads to better disease prediction and explanation. The contributions of this work are as follows:

- The Random Oversampling technique is part of data balancing in the cardiovascular dataset.
- A novel CatBoost-BEEs hybrid model is presented that accurately identifies heart diseases in their early stages. We used the BEE algorithm to optimize the hyperparameters of the CatBoost model.
- XAI technique, SHAP as a global explanation, and LIME technique are used as a local explanation to highlight key features influencing cardiac conditions in the proposed CatBoost-BEE model, offering valuable insights that can support CVD decision-making and improve patient care.

The paper includes an extensive literature review summarizing the studies relevant to the proposed work. Then, a detailed description of the materials and methods is elucidated, which describes the dataset and explains the process involved in analysing the dataset. Following this section, the results are discussed, emphasizing the outcomes generated from the project and presenting a comparative analysis that justifies the unique contribution of the work conducted. Finally, the Conclusion Section wraps up the work by outlining the key findings and suggesting avenues for further investigation.

### Literature review

Machine learning (ML) methods are increasingly being used to identify cardiovascular diseases, with numerous studies examining various machine learning algorithms and deep learning models. This method may revolutionise healthcare by enabling more precise diagnoses and individualised patient treatment plans. This segment overviews previous research using ML approaches for CVD identification. In one study^8^, the authors introduced a Random Forest (RF) algorithm that explores the correlation between heart disease and diabetes. This technique estimates the percentage of heart disease risk, taking into account the impact of diabetes on coronary artery disease. The study suggests that better results can be achieved by incorporating additional parameters. Another study^1^ focused on using healthcare data to predict CVD via a mobile iPhone Operating System (iOS) application, achieving 72% accuracy. The authors suggest extending this model to include other diseases for further performance improvements. In^9^, four classification methods—K-Nearest Neighbors (KNN), Decision Trees (DT), Naïve Bayes (NB), and RF—were used to propose a model. The study employed various data mining techniques to glean insightful information from massive datasets, including association rules, clustering, and regression. KNN yielded the maximum accuracy. According to the authors, utilising more sophisticated models and additional methods, including time-series analysis, integrated association and clustering criteria, Support Vector Machines (SVM), and evolutionary algorithms, may enhance the accuracy of CVD prediction. In another study^10^, the authors applied DT, NB, and Neural Networks to develop an Intelligent Heart Disease Prediction System (IHDPS), which improved accuracy and also reduced costs and time. Their findings showed accuracy rates of 60% for NB, 61.45% for Logistic Regression (LR), and 64.4% for SVM. In^11^, the researchers worked on enhancing coronary artery disease prediction by using effective techniques like the Hoeffding Tree (HT), Gaussian NB, RF, SVM, and Logistic Model Tree (LMT). Among these, RF yielded the most accurate results. They suggested adding more features and reducing data size, which could further improve the performance of the genetic algorithm. In^12^, the authors presented a cardiac disease prediction method using Python-based libraries, including scikit-learn (sklearn), pandas, and TkInter, achieving 88% accuracy with a hybrid model. The study proposes that Deep Learning (DL) techniques could lead to even better predictive outcomes for heart disease. In this paper^13^, the authors propose a Hybrid Deep Learning (HDL) model named DenseNet-ABiLSTM for multiclass arrhythmia detection and classification using Photoplethysmography (PPG) signals, supplemented with ECG data. The goal is to classify six types of arrhythmias: Sinus Rhythm (SR), Early Ventricular Contraction (EVC), Early Atrial Contraction (EAC), Ventricular Tachycardia (VT), Supraventricular Tachycardia (ST), and Atrial Fibrillation (AF). The method was evaluated on 109,736 PPG-ECG segment pairs collected from 225 patients. The DenseNet-ABiLSTM model achieved 89.14% of Mean Accuracy, 87.74% of Mean F1 Score, and 98% of ROC AUC. The primary challenge is accurately classifying arrhythmias from noisy PPG signals, particularly with class imbalance and overlapping patterns in similar or underrepresented types, such as VT and AF. The authors in^14^ analysed CVD prediction using ML techniques. Random Forest (RF) achieved an accuracy of 95.60% among the algorithms evaluated. This study suggests that further improvements could be made by incorporating other algorithms and advanced deep-learning methods. In^15^, the researchers developed a method to assess and summarize the predictive capabilities of ML techniques in heart disease prediction. They found that algorithms such as SVM and Boosting Algorithms (BA) show promising results for CVD prediction. By utilising additional feature selection techniques, accuracy can be enhanced. In^16^, an ML model for forecasting cardiovascular disease, incorporating a neural network-based framework, will be introduced. Their model achieved an accuracy of 85.71%, and they suggested that increasing the size of the testing datasets could further improve performance. In a study^17^, created a CVD prediction approach utilizing Naïve Bayes (NB) and Decision Tree (DT) classification algorithms. While the Decision Tree algorithm initially outperformed Naïve Bayes, it removed inconsequential features to improve NB’s accuracy, highlighting the importance of pre-processing for effective heart disease prediction. In^4^, this approach for predicting cardiovascular disease, six ML algorithms were used. SVM and Multilayer Perceptron (MLP) attained the highest accuracy of 91.7%, and the study suggests that exploring ensemble methods and fine-tuning parameters could enhance the results further. This work^18^ proposes hybrid ensemble machine learning approaches using the Cleveland heart disease dataset, employing Extreme Gradient Boosting (XGBoost), Adaptive Boosting (AdaBoost), Gradient Boosting (GB), Light GB Machine (LightGBM), CatBoost, and Histogram-Based Gradient Boosting as classification algorithms. The Meta-XGBoost and Meta-CatBoost obtained amazing accuracy of 93.37% and 93.28%, respectively. The study^19^ uses CatBoost, XGBoost, Gradient Boosting, Multilayer Perceptron, Random Forest, Naïve Bayes, Decision Tree, k-Nearest Neighbor, AdaBoost, and SVM-Sigmoid models. Among them, the CatBoost model comes within 86.9% of the ROC curve with 100% specificity and precision to predict CVD. This paper^20^ tests RF, DT, CatBoost, XGBoost, GBoost, LightGBM, and AdaBoost algorithms to predict CVD, with CatBoost demonstrating 100% specificity and precision. This work suggests incorporating correlation feature-based selection to improve the predictive ability of boosting classifiers for heart disease. In this study^21^, to predict heart failure, they used Logistic regression (LR), SVM, KNN, Gaussian Naïve Bayes (GNB), and Multinomial Naïve Bayes (MNB) as Linear Machine Learning Models. Random forests and gradient boosting classifiers, AdaBoost, Bagging trees (BT), Extra trees (ET), and Decision trees (DT) as Ensemble Learning Models. CatBoost, XGB, LGBM, and Hist. Gradient Boosting Classifiers (Hist. GBC) are Boosting Classifiers. CatBoost achieves a higher accuracy of 87.93%. They believe CatBoost, random forests, and gradient boosting, in conjunction with other predictive algorithms, are ideal for forecasting the occurrence of heart disease. This study^22^ used five prediction models (SGB, MARS, Lasso, Ridge, and CatBoost) to predict the early stage of hypertension. CatBoost provided 68.2% of Sensitivity, 61.3% of Specificity, and 70.3% of AUC. To find^23^ coronary artery disease (CAD), CatBoost is used with an accuracy of 74.78%, a Sensitivity of 67.80%, a Specificity of 80.20%, and an F1 score of 69.83%. This area undoubtedly merits further investigation to examine new techniques to improve model explainability in future work. In this paper^24^, after using the BEE optimization algorithm (BEE) to determine the best subset of features from the complete collection in the CVD prediction dataset, the false negative rate decreased in the Random Forest model, and the accuracy increased. Overall, the existing literature on cardiovascular disease detection through Machine Learning algorithms exhibits that although significant advancements have been made, there is still room for improvement. Many studies have focused on classification and prediction, but research on more efficient data pre-processing methods to improve algorithm performance remains limited. While some models have gained high accuracy, there is still a need for more precise early-stage CVD prediction and identification of reliable risk indicators. Additionally, studies utilizing deep learning (DL) methods on tabular data for CVD detection are scarce. Most research relies on limited datasets, raising concerns about the generalizability of the findings. Furthermore, explainable AI and data balancing methods have been underexplored, even though these methods can provide valuable insights into model performance. By creating a CatBoost Bees hybrid, more effective and efficient machine learning models for CVD prediction, this work seeks to close these gaps. In a tabulated format, surveys of limitations, methods, and results of some recent works are included in Table 1.

**Table 1:** Survey of some recent work on CVD based on our proposed work (RSS: Random Subspace Sampling, Novel CapNet: CapNet -based model with Gaussian Optimization, M2MASC-enabled CNN-BiLSTM**:** Modified Mixed Attention-enabled Search Optimizer-based Convolutional Neural Network–Bidirectional Long Short-Term Memory, SCN: Signal Classification Network or Spatio-Temporal Convolutional Network).

Refs.	Methods	Matrices & results	Key challenges
^1^	XGBoost	Accuracy-72.70%, F1-score-72%	Model accuracy is modest; it lacks XAI for interpretability; application is optimized for iOS only—performance may degrade on Android; future improvements suggested include deep learning and CNN models to surpass 90% accuracy
^4^	SVM, MLP	Accuracy-91.7%, 91.7%; Sensitivity-91.7%, 95.8%; Specificity-91.7%, 88.9%; AUC-96.8%, 93.6%	No explainability tools were integrated; a small sample size of 300 limits generalizability; only a single dataset was used; some features with missing values were dropped rather than imputed, which could affect model robustness
^8^	RF	Accuracy-84.81%	XAI was not applied; the model is limited to structured tabular datasets, and performance may vary depending on pre-processing and class distribution; accuracy still leaves room for improvement, and only binary classification was considered
^9^	K-NN	Accuracy-78.95%	Limited to a small, single dataset; no XAI for interpretability; accuracy affected by attribute selection and missing value handling; potential improvement exists by combining models or using advanced techniques like SVM or neural networks
^10^	SVM	Accuracy-91.67%, Precision-92.31%, Recall-88.89%, F1-score-90.56%	With a limited dataset, no dedicated XAI, and reliance on feature selection and pre-processing, generalization may suffer without diverse data
^11^	RF, SVM, NB, HT, LMT	Accuracy-95.08%, 90.16%, 93.44%, 81.24%, 80.69%	No XAI used, small single dataset, basic metrics, and possible overfitting despite ensemble tuning; broader validation needed for generalization
^12^	Hybrid model (DT + RF)	Accuracy-88%	No XAI used, moderate dataset limits generalization; performance depends on feature quality; future work suggests deep learning for better results and multiclass classification
^14^	RF	Accuracy-95.60%, Precision-55.28%, Recall-97.68%	XAI was not used; some algorithms had high computational cost, with MLP and Deep Neural Network prone to overfitting and performance varying by dataset size and feature complexity
^15^	SVM, BA,	Sensitivity-57.0%, 85% Specificity-93%, 85% AUC-92%, 91%	The study notes limitations like high heterogeneity from inconsistent algorithm reporting, lack of standard metrics, unclear feature selection, small subgroup samples, inconsistent clinical alignment, and absence of external validation and model transparency
^16^	RF	Accuracy-85.71% ROC-86.75% AUC-6.75%	Though not stated explicitly, limitations include a small dataset (304 samples), no external validation, no class imbalance handling, and the absence of XAI, affecting transparency and clinical trust
^18^	Ensemble model	Accuracy-94.76%, Precision-94%-95%, Recall-93%-96%, F1-score-93%-95%	Limitations include no XAI, a single dataset without external validation, risk of overfitting, and no real-world clinical testing. Future work aims to add remote monitoring and more algorithms for better performance
^19^	CatBoost	Accuracy-89.5%, Precision-82.7%, Recall-85.6%, F1-score-83.6%, AUC-86.7%	The model lacks effects of newer medications and visit-to-visit clinical variability, which are planned for future updates to enhance prediction
^20^	AdaBoost	Accuracy-95.2%, Recall-95.2%, Specificity-95.3%, Precision-98.7%, F1-score-96.98%, FPR-4.69%, FNR-4.75%	The paper does not explicitly state limitations but implies potential improvements such as using larger primary datasets, developing a web application for real-time prediction, integrating deep learning methods, and incorporating image data for future enhancements
^21^	CatBoost	Accuracy-87.93%	The paper implies limitations like a lack of interpretability, limited generalizability due to a small dataset, and no external validation, with future improvements suggested via survival prediction and real-time sensor integration
^22^	CatBoost	Accuracy-69.8%, Sensitivity-71.3%, Specificity-80.9%, AUC-76.4%	The study’s limitations include using a single Taiwanese dataset, no longitudinal follow-up or causal inference, untested generalizability to clinical populations, and exclusion of elderly or chronically medicated individuals. It highlights the need for future cohort-based validation to confirm clinical utility
^23^	CatBoost	Accuracy-78.82%, Sensitivity-74.63%, Specificity-82.05%, F1-score-75.32%	Limitations include the absence of imaging data, regional data bias, and the exclusion of key risk factors like dyslipidemia, affecting generalizability
^24^	Hybrid (SVM + BEE)	Accuracy-85.85%, Recall-84.98%, Specificity-86.97%, FPR-15.11%, FNR-13.03%	Limitations include a small dataset, no external validation, and a lack of interpretability; future work should use larger datasets and more features for better prediction
^25^	M2MASC-enabled CNN-BiLSTM	Accuracy-98.25%, Precision-99.57%, Recall-97.53%	The suggested model is fairly accurate and secure but has several problems. It depends a lot on IoT and the cloud, costs a lot to compute, is sensitive to noisy signals, has a lot of blockchain overhead, and it’s unclear how well it will work in the real world
^26^	M2AM–Deep BiLSTM	Accuracy-93.82%, Precision-96.20%, Recall-94.34%	The model exhibits accuracy; nevertheless, it is constrained by substantial computing expenses, diminished interpretability, reliance on specific datasets, absence of clinical validation, privacy issues, and restricted illness coverage
^27^	SCN–Deep BiLSTM	Accuracy-97%, Precision-98%, Recall-99% F1-score-97%	The model is accurate but limited by high computational cost, risk of overfitting, dataset dependency, low interpretability, scalability challenges, and lack of real-world clinical validation
^28^	Probabilistic Neural Network (PNN)	F-score: 99.83% Sensitivity: 99.54%	The proposed method performs well but faces challenges such as high computational cost, complex parameter tuning, class imbalance, PNN sensitivity, and limited generalizability due to single-lead ECG use. No XAI used
^29^	Improved Hawks Optimizer	Accuracy-97%, Precision-98%, F1-score-97%, Specificity-96%, FPR-3.89%, FNR-2.15%	The paper tackles major issues in heart disease prediction, such as data imbalance, outliers, and weak optimization, by introducing an improved Hawks Optimization-based ensemble model that enhances feature selection and prediction performance. No XAI used
^30^	Ensemble Model(K-NN,XGBoost,AdaBoost,RSS)	Accuracy-96.2%, Precision-97.2%, Recall-95.2 F1-score-96%,	Data restrictions, feature inconsistency, and high dimensionality cause overfitting and poor heart disease prediction using machine learning. Preprocessing, hybrid feature selection, and ensemble methods are needed to improve model accuracy and dependability
^31^	Novel CapNet	Accuracy-95%, Precision-94% Sensitivity-97%, Specificity-94%, F1-score-95%	The study tackles model performance issues from biased training data by isolating such subsets using VAE and PCA, and improves prediction through optimized feature selection and network tuning. No XAI used

## Materials and methods

**Fig. 1:**
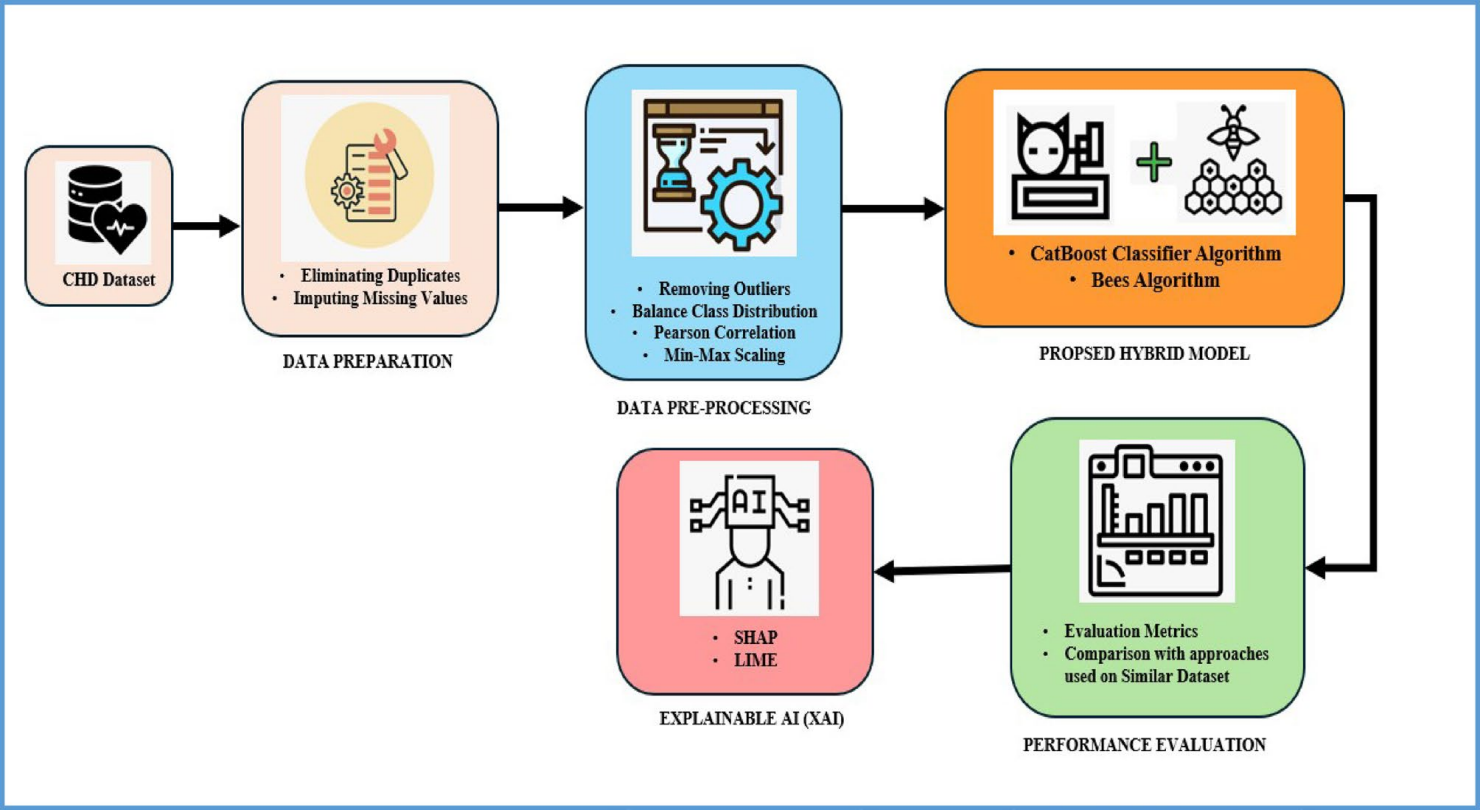
Architecture diagram of the proposed framework.

Figure 1 presents the architecture of the proposed method, which includes data collection, data preparation, pre-processing, hybrid model design for hyperparameter optimization, model evaluation, comparison with state-of-the-art approaches conducted on a similar dataset, and finally, an explanation about the justification of the acquired output.

### Data description

The study used a cardiovascular dataset based on contemporary cardiovascular research performed on citizens in Framingham, Massachusetts, publicly available on the Kaggle website[https://www.kaggle.com/datasets/aasheesh200/framingham-heart-study-dataset]^32^. The dataset included categorization emphasizing patients’ 10-year potential risk of developing cardiovascular disease (CVD). The dataset comprised 4238 recordings, each containing 16 significant attributes related to the occurrence of CVD. The attributes included behavioural, medical, and demographic aspects, all of which were substantial risk factors for the occurrence of CVD, as shown in Table 2.

**Table 2:** Data description.

Sl. no	Features name	Description
1	Gender	Male represents 1, and female represents 0
2	Age	The patient’s age continuous value
3	Education	Highest degree of patient, categorical data (1 for school,2 for college,3 for university,4 for higher)
4	Current smoker	If a person has smoked, then 1 otherwise 0
5	Cigs per day	How many cigarettes are smoked per day?
6	BP meds	The person was on blood pressure medication. no = 0, yes = 1
7	Prevalent stroke	The person had a stroke in the past. no = 0, yes = 1
8	Prevalent hypertensive	The person was hypertensive or not. no = 0, yes = 1
9	Diabetes	If a person had diabetes = 1 or = 0
10	Tot Chol	Patient cholesterol level (continuous)
11	Sys BP	Systolic blood pressure (continuous)
12	Día BP	Diastolic blood pressure (continuous)
13	BMI	Body mass index (continuous)
14	Heart rate	Heart rate (continuous)
15	Glucose	Glucose level (continuous)
16	Target	10-year risk of coronary heart disease CHD (binary: “1”, means “Yes”, “0” means “No”)

### Date preparation

**Fig. 2:**
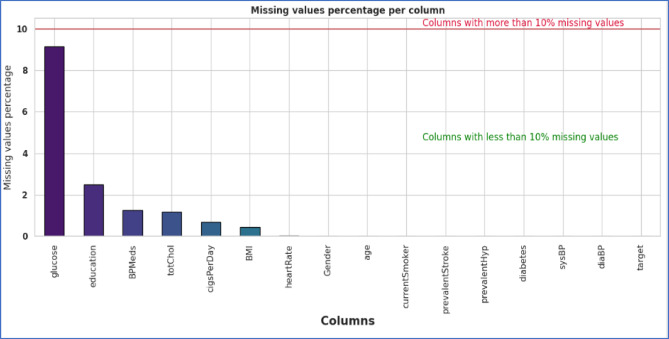
Missing value of the Framingham CVD dataset.

As part of the data preparation process, which is highly critical in ML, it is ensured that the data used in the study is accurate, clean, and formatted, which would contribute towards the performance of the ML model and in generating promising insights from the derived outcome. In this regard, some changes were made to the “10-year risk of coronary heart disease CHD” features, being labelled as “Target” and including the label “Gender” as a feature in the dataset. The dataset contained no duplicate values; however, a systematic approach was employed for data preparation, involving the imputation of missing values using the Median Imputation technique^33^. Figure 2 highlights the missing values eliminated using the Median Imputation Technique. The Median imputation technique uses central tendency measures in a dataset to fill in the missing values. The mathematical formula for this technique is shown in Eq. (1)

1 $$ \hat{x}_{median} = Median\left( {x_{1} ,x_{2} ,x_{3} \ldots x_{n} } \right) $$

where: ${x}_{i}$ = Non-missing values in the variable; n = Number of non-missing values.

We replaced missing values with the mean for continuous features “cigsPerDay” = 29, “totchol” = 50, “BMI” = 19, “heartrate” = 1, “glucose” = 388, and the median for categorical features “education” = 105, and the mode for binary features “BPMeds” = 53.

### Data pre-processing

As part of data pre-processing, an outlier handling technique—Box plot generation was used, followed by the Random Oversampling (RO) technique for data balancing, the Pearson correlation technique, and the Min–max scaling technique. The use of these techniques enabled the removal of outliers from continuous features. Furthermore, it enhanced the model’s performance, reducing the likelihood of misleading conclusions resulting from Measurement Errors, Data Entry Errors, Natural Variation, Sampling Errors, and other sources.

### Outlier handling

**Fig. 3:**
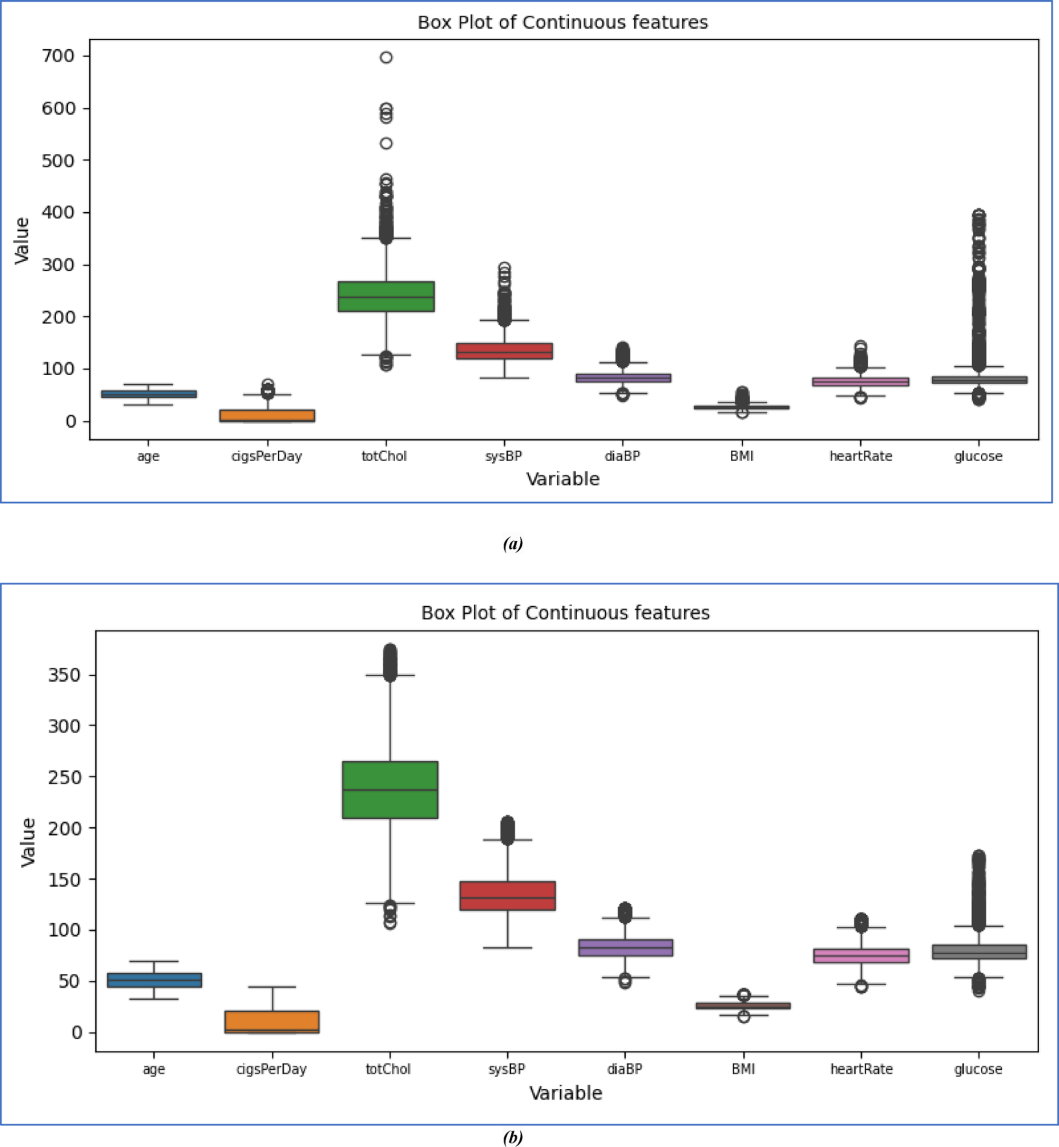
Box plot of continuous features to view outliers. (**a**) represents the Box plot before removing outliers, and (**b**) represents the Box plot after removing outliers.

Outliers are data points that significantly deviate from the majority of observations in a dataset. It can skew statistical analyses, affect model performance, and lead to misleading conclusions. Outliers may occur due to measurement errors, Data Entry Errors, Natural Variation, Sampling Errors, etc. Here is our study. We used a box Plot shown in Fig. 3 to identify outliers, and we removed all outliers from all continuous features based on the Z-score method^34^, where the Z value is in the range of -3 < Z > 3, which comes under a 95% confidence interval.

### Balancing class distributions

**Fig. 4:**
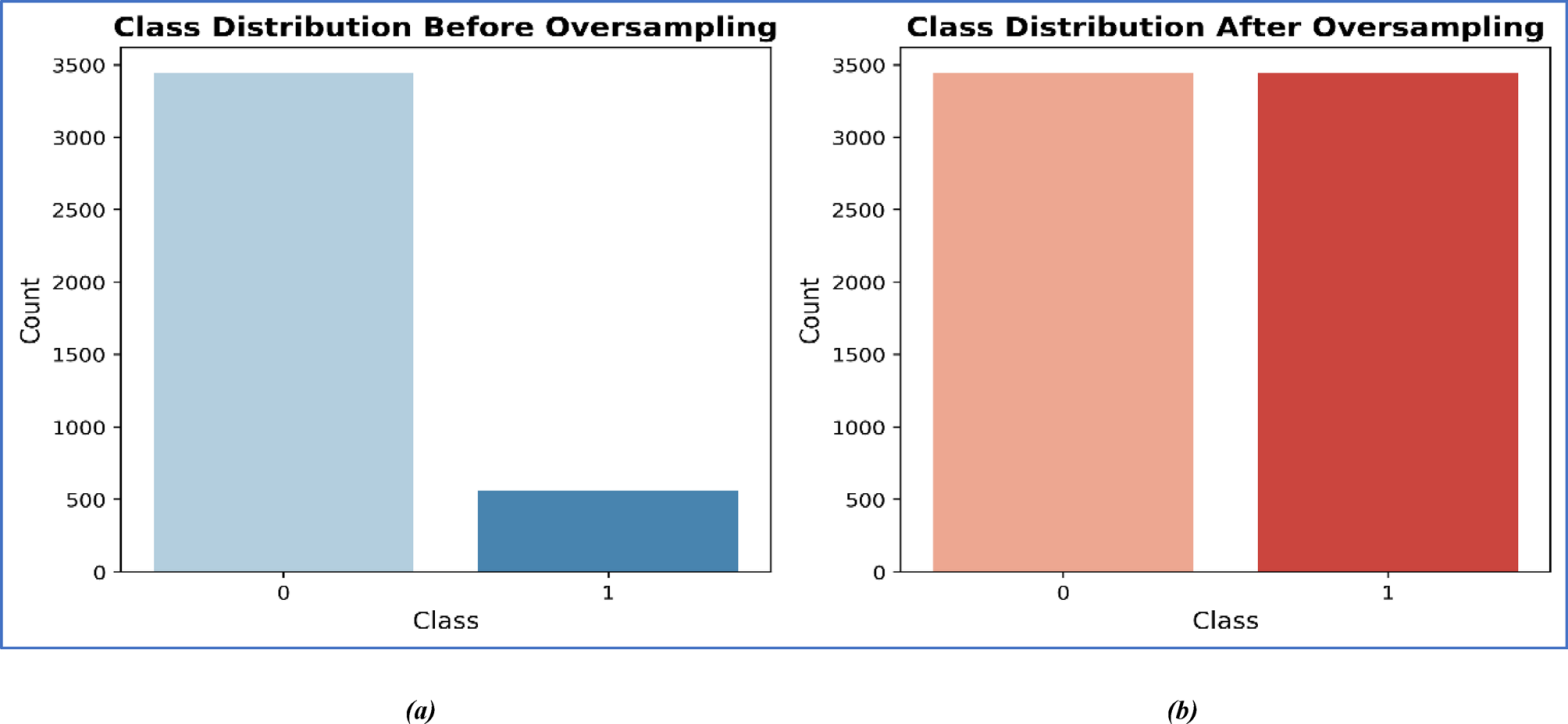
The target variable of the dataset. (**a**) is before applying RO, and (**b**) is after applying RO. Class 0 indicates no CVD, and Class 1 indicates CVD.

Balancing class distributions is crucial in machine learning when dealing with imbalanced datasets, where one class is overrepresented compared to others, as shown in Fig. 4a. Here, we used the Resampling Techniques technique to address this problem. By interpolating between pre-existing samples, we created synthetic samples using the Random Oversampling (RO), which prevents the possibility of overfitting due to sample duplication^35,36^. After applying RO, the resultant dataset is reflected in Fig. 4b.

### Features selections (correlation)

**Fig. 5:**
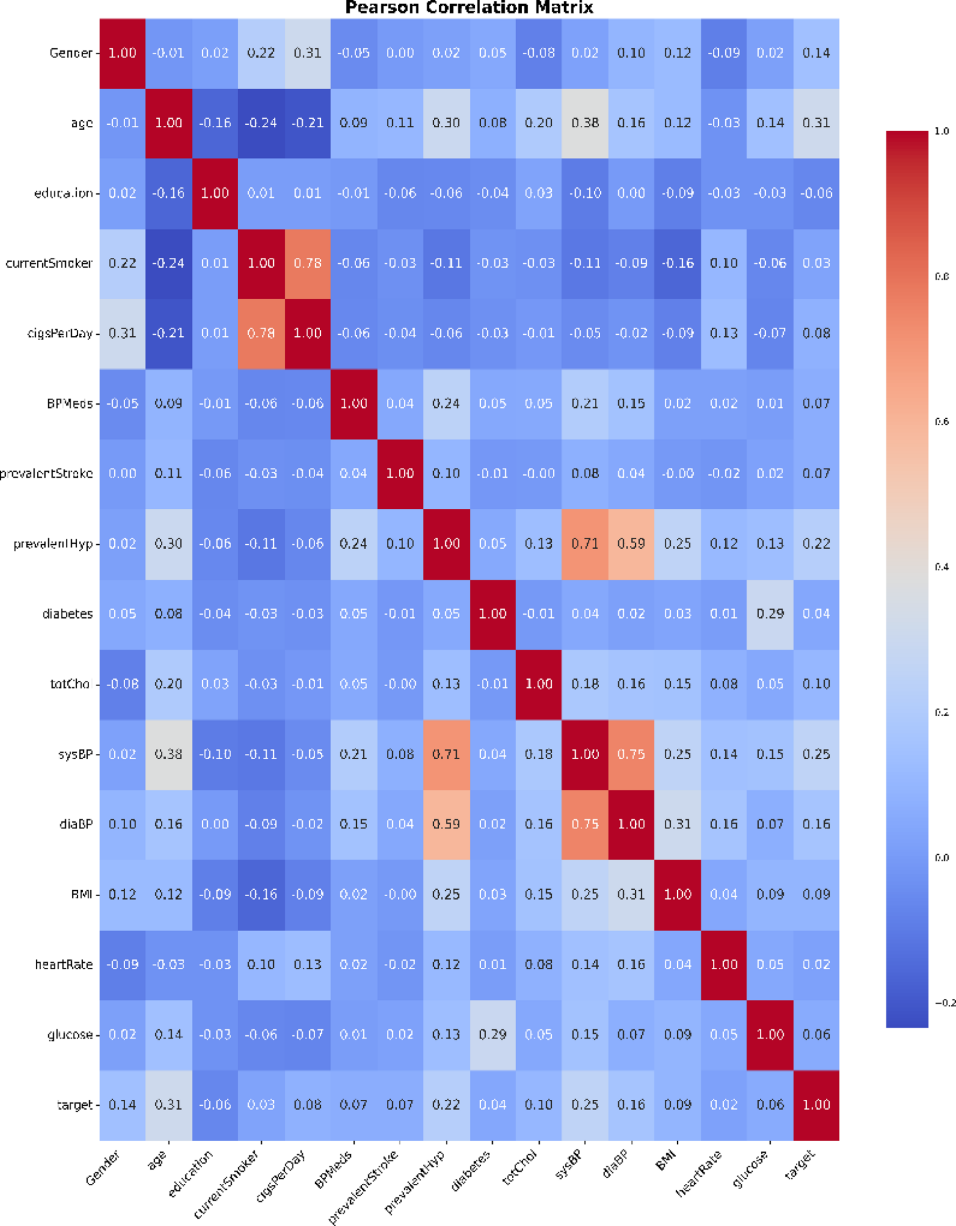
Heat map of correlation of features.

Correlation is a statistical term representing the amount and direction of a relationship between two variables. In data science and machine learning, correlation helps us understand how one variable changes in relation to another and can be instrumental in feature selection, data analysis, and hypothesis testing. It assesses the degree and direction of the link between the variables. A Pearson correlation^37^ analysis was conducted, which quantified the relationship between the two variables. The threshold value observed for the correlation coefficient in this study was 7.9. Also, exploratory data analysis (EDA) was performed to understand the data characteristics, and the findings are presented. Figure 5 illustrates a heatmap that highlights inter-feature correlations and their associated values. The coloured cells represent the correlation between two features, with distinct Colours indicating negative correlations and zero indicating no correlation.

### Scaling data

Scaling is a crucial step that adjusts the range or distribution of features in a dataset, making them easier for models to interpret and improving their performance. Efficiency of Optimization Algorithms. Scaling ensures that hole features are available equally to the model and prevents variables with huge magnitudes from overwhelming those with lower ones. For our study, the Min–Max scaling technique^38^ was used for up to four decimal values as part of data normalization, converting the values of the features to the pre-determined range between 0 and 1. The equation presenting the mathematical formula used for the Min–Max scaling technique is shown in Eq. (2) The scaling technique enables improved interpretation, performance, and efficiency of the optimized algorithms, preventing the variables with enormous magnitude from overwhelming the lower values.

For a given feature x, each value ${x}_{\mathrm{i}}$ is transformed as follows:

2 $$ x^{\prime} = \frac{{x_{{\mathrm{i}}} - x_{{{\mathrm{min}}}} }}{{x_{{{\mathrm{max}}}} - x_{{{\mathrm{min}}}} }} $$

where:

- $x_{{\mathrm{i}}}$: Original value of the feature
- $x_{{{\mathrm{min}}}}$: Minimum value in the feature
- $x_{{{\mathrm{max}}}}$: Maximum value in the feature
- $x^{\prime}$: Scaled value in the range [0, 1]

### Data splitting

It is pertinent to mention that the dataset is divided in a ratio of 4:1, wherein 80% of the data is used in training and 20% is used for testing.

## Methodology

Bio-inspired Optimization Algorithms mimic biological processes, such as the behaviour of animals, evolution, or ecosystems, to find optimal solutions in complex search spaces. These algorithms are commonly used for engineering, computer science, and finance optimization tasks, especially when traditional methods struggle with high-dimensional, non-linear, or multi-modal problems. Bio-inspired algorithms are valuable for complex optimization problems due to their adaptability, ability to escape local minima, and their natural exploration–exploitation balance. These algorithms are particularly beneficial for high-dimensional, non-linear, and multi-modal optimisation tasks, where they often outperform traditional optimisation methods. In this study, we used Particle Swarm Optimization (PSO)^39^, Grey Wolf Optimizer (GWO)^40^, Cuckoo Search Algorithm (CSA)^41^, and the Bees Algorithm (BEE)^42^ as bio-inspired optimization algorithms to optimize hyperparameters of the Categorical Boosting (CatBoost) Classifier^43^, and we got the best result in the Bees Algorithm-CatBoost, which we consider our proposed hybrid model: the Bees Algorithm, a population–based search algorithm reflecting the food searching behavior of the honeybees. The algorithm performs local search in combination with global search and contributes towards continuous optimization. The CatBoost classifier is a gradient-boosting algorithm that excels at handling categorical values and achieves optimised accuracy. The integration of the Bees algorithm with CatBoost is used in this study to create our proposed hybrid model, leading to the generation of an optimized outcome, as reflected in the evaluation metrics of the proposed model. Then, the outcome explanation is provided through Shap and Lime for both global and local explanations.

### Proposed model (Hybrid CatBoost-the Bees algorithm framework)

The proposed hybrid framework combines CatBoost, a Categorical Gradient Boosting Classifier, with the BEE to automatically determine the optimal values for its most critical hyperparameters. The primary objective is to enhance the model’s predictive performance by minimising its error through an efficient search of the hyperparameter space, inspired by the intelligent foraging behaviour of bees.

1. Objective function

The CatBoost model performance depends on a set of hyperparameters (α, d, T, s, λ) shown in Eq. (3):

3 $$ F_{T} \left( x \right) = F_{0} \left( x \right) + \alpha \mathop \sum \limits_{t = 1}^{T} \frac{1}{1 + \lambda }h_{t} \left( {x;d,s} \right) $$

Keys:

- Learning rate $(\alpha )$: Controls how quickly the CatBoost model learns from errors.
- Maximum tree depth $(d)$: Determines the complexity of individual trees of the CatBoost model.
- Number of trees $(T)$: The total number of decision trees used in the CatBoost model (Iterations).
- Subsample ratio $(s)$: Fraction of data used for training each tree to prevent overfitting of the CatBoost model.
- L2 Regularization coefficient (λ): Penalizes large weights to reduce overfitting of the CatBoost model.
- ${F}_{T}\left(x\right)$ : Final CatBoost model prediction after T trees.
- ${F}_{0}\left(x\right)$: Initial CatBoost model prediction.

The optimization goal is to minimize the loss between the actual target values $y$ and the predicted values $\widehat{y}$ generated by CatBoost, and $L()$ is the chosen loss function, such as cross-entropy for classification or mean squared error for regression, is defined as in Eq. (4):

4 $$ f\left( {\alpha ,d,T,s,{\uplambda }} \right) = L\left( {y,\hat{y}} \right) $$

2. Bees algorithm–based optimization

In this hybrid model, each bee represents a candidate hyperparameter vector (α, d, T, s, λ). The algorithm refines these candidates through an iterative process of exploration and exploitation.

*Step 1*: **Initialization**

- Initialize $\:N$ scout bees with random hyperparameters $\:f\left(\alpha\:,d,T,s,{\uplambda\:}\right)$ in a feasible range.

*Step 2*: **Employed Bees Phase**

- Each scout bee trains a CatBoost model with its set of hyperparameters and evaluates $\:f\left(\alpha\:,d,T,s,{\uplambda\:}\right)$.
- The top-performing solutions are retained, and the positions (hyperparameters) are shared with onlooker bees.

*Step 3*: **Onlooker Bees Phase**

- Onlooker bees exploit the best positions found by employed bees by conducting local searches around these hyperparameter values.

*Step 4*: **Scout Bees Phase**

- Any positions (hyperparameter sets) that do not improve after $\:iterations$ are abandoned, and new random positions are assigned to the corresponding scout bees.

*Step 5*: **Update and Convergence**

- This process repeats until the change in $\:f\left(\alpha\:,d,T,s,{\uplambda\:}\right)$ falls below a specified threshold or a maximum number of iterations is reached.

*Step 6*: **Best Hyperparameters**

- The hyperparameter set with the lowest objective function value is selected to configure the CatBoost model. This hybrid method can lead to a well-tuned CatBoost model, achieving potentially higher accuracy than standard grid or random search approaches, as the Bee Algorithm’s intelligent search strategy efficiently explores the hyperparameter space.

This hybrid technique thus leads to a well-tuned CatBoost model, achieving higher accuracy than standard grid or random search approaches because the Bee Algorithm’s intelligent search strategy efficiently explores the hyperparameter space.

### Explainable Artificial Intelligence (XAI)

Explainable Artificial Intelligence (XAI) is a set of methods that enable humans to achieve interpretability and trust in the results generated from machine learning models. It helps to provide explainability to the AI models and understand their expected impact and associated biases. Traditional ML models often produce results that are difficult to comprehend and understand the attributes that significantly contributed to the algorithms’ outcomes. Hence, the entire process becomes a “black box,” making it impossible to retrace or interpret the results. Using XAI helps users gain confidence in using AI models and adopt the approach for real-time implementation.

**LIME** (Local Interpretable Model-agnostic Explanations)^44,45^ is a technique used to explain black-box machine learning models by approximating them locally with interpretable models. It is model-agnostic and can be used with any machine learning model. LIME focuses on explaining individual predictions by approximating the decision boundary of the complex model in the local region around a specific data point. LIME thus bridges the gap between complex, black-box models and human-understandable interpretations by creating locally faithful, interpretable models around individual predictions.

**SHAP** (Shapley Additive Explanations)^46^ is a powerful paradigm for assessing machine learning models that employ Shapley values from game theory. It provides a fair and consistent way to express how each characteristic influences model predictions. Explains both individual forecasts and the overall relevance of features. Shapley values are used to guarantee that each feature contributes proportionally. Works on any model. It supports tree-based models, deep learning, and linear models. SHAP scores reflect how much each attribute helps or hurts a prediction. Increases trust and transparency by letting people know what variables impact AI model decisions.

## Results

This investigation was carried out on a laptop with a Windows 10 operating system, an Intel Core i5 CPU, and 8 GB of RAM. The experiments were performed using Google Colab. Among the four CatBoost-associated bio-inspired optimization algorithms—Particle Swarm Optimization (PSO)**,** Grey Wolf Optimizer (GWO)**,** Cuckoo Search Algorithm (CSA), and the Bees Algorithm (BEE)**—**our proposed hybrid model using the Bees Algorithm achieved the highest classification accuracy of 98.04%**.** The performance comparison of all four models is presented in Table 3, while the **optimized hyperparameters** selected by the Bees Algorithm for our proposed model are detailed in Table 4.

**Table 3:** Accuracy comparison of different bio-inspired optimization algorithms associated with CatBoost.

Optimization algorithms	Accuracy (%)
Particle Swarm Optimization (PSO)	97.53
Grey Wolf Optimizer (GWO)	97.75
Cuckoo Search Algorithm (CSA)	96.95
Bees Algorithm (BEE)	98.04

**Table 4:** Selected values of hyperparameters.

Hyper parameter	Value
Learning rate $(\alpha )$	0.1197
Maximum tree depth $(d)$	12
Number of trees $(T)$	631
Subsample ratios $(s)$	1
L2 Regularization coefficient (λ)	3

**Fig. 6:**
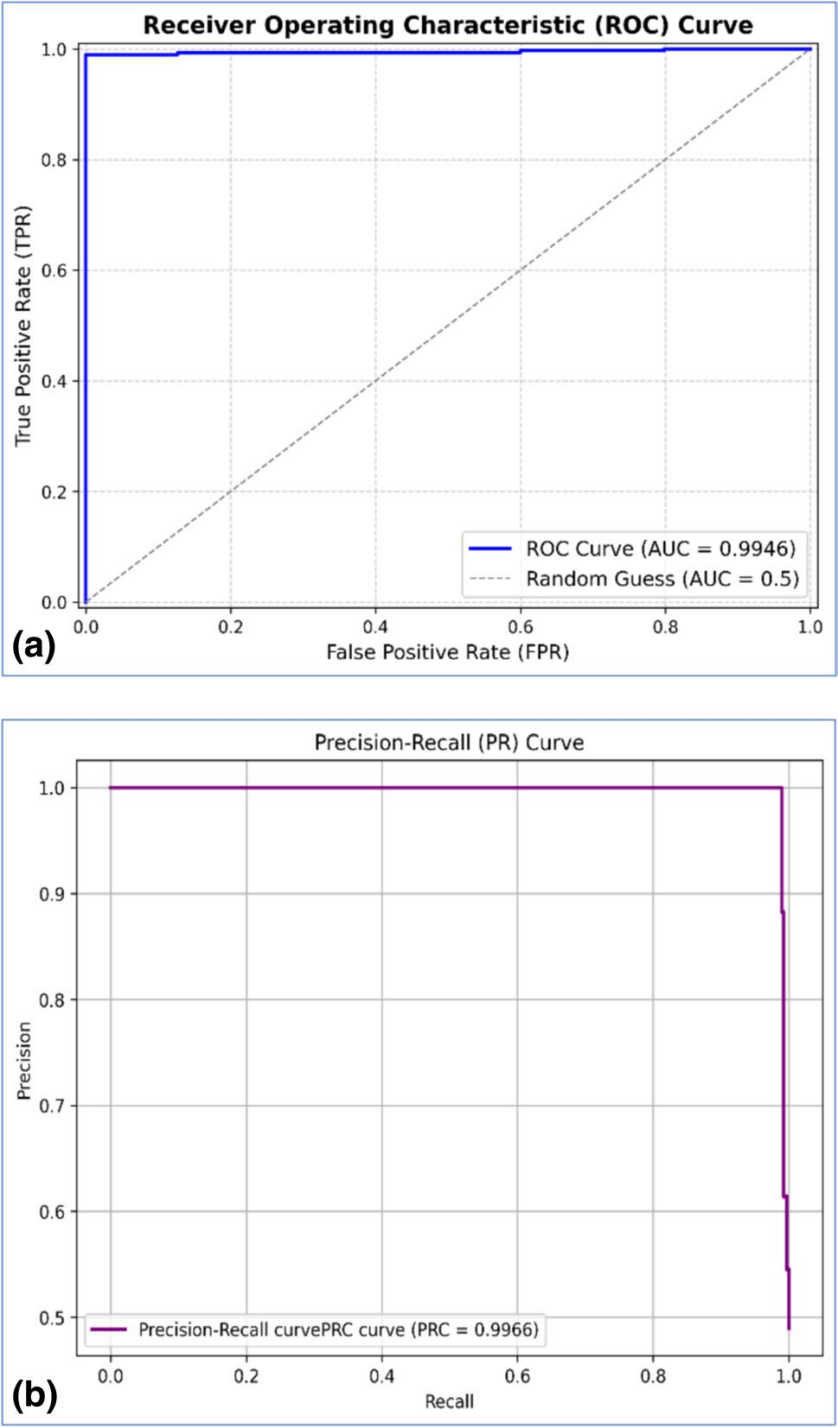
(**a**, **b**) represent the ROC curve and PRC curve of the proposed model, respectively.

The proposed model exhibited promising outcomes, as reflected in the evaluation metrics’ results. Tables 5 and 6. Highlights the superiority of the proposed model. The proposed model yielded an accuracy of 98.02%, a Precision of 97.09%, a Recall of 98.96%, an F1-score of 98.02%, a Specificity of 97.16%, a False positive rate of 2.84%, a False negative rate of 1.04% and an MCC of 96.04%. The area under the ROC (Receiver Operating Characteristic) curve signifies the common region between the actual positive and false favourable rates. In contrast, the area under the PRC (Precision-Recall Curve) illustrates the common area between precision and recall. The Proposed hybrid model achieved 99.45% of ROC and 99.65% of PRC, respectively. Figure 6a, b illustrate the ROC curves and PRC curves for a graphical view of classification methods, respectively.

**Table 5:** Evaluation matrices of the proposed model.

Proposed model	
Accuracy	0.9804
Precision	0.9709
Recall	0.9896
F1-score	0.9802
Specificity	0.9716
FPR	0.0284
FNR	0.0104
MCC	0.9604
Cohen’s Kappa score	0.9608
ROC	0.9945
PRC	0.9965
Support	1379
Total time (Execution time + Prediction time)	26.0580 (sec.)

**Table 6:** Classification report of the proposed model.

	Precision	Recall	F1-score	Support
Class 0	0.99	0.97	0.98	704
Class 1	0.97	0.99	0.98	675
Macro average	0.98	0.98	0.85	1379
Weighted average	0.98	0.98	0.98	1379

**Fig. 7:**
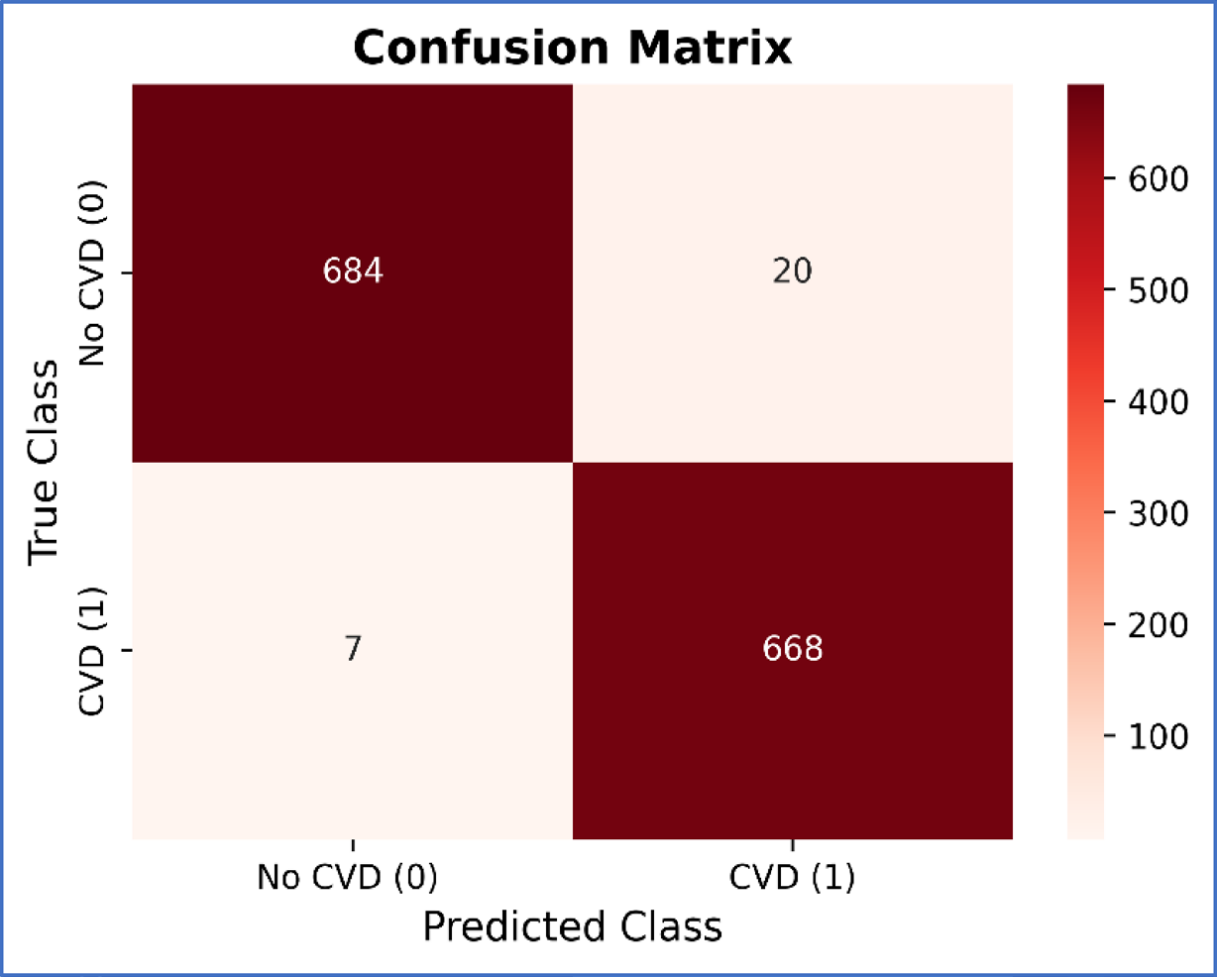
Confusion matrix of the proposed model.

**Fig. 8:**
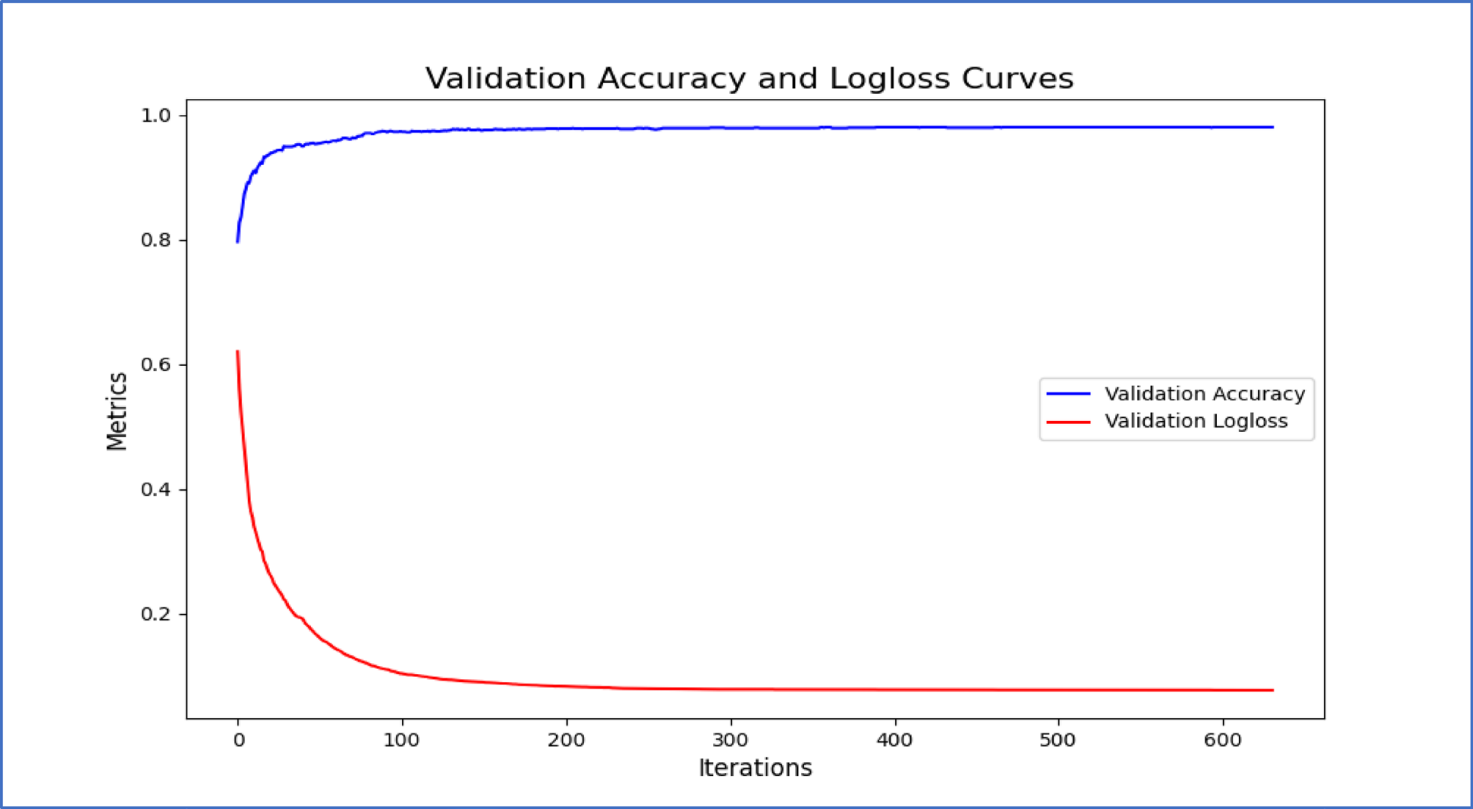
Accuracy vs. Loss curve of the proposed model.

**Fig. 9:**
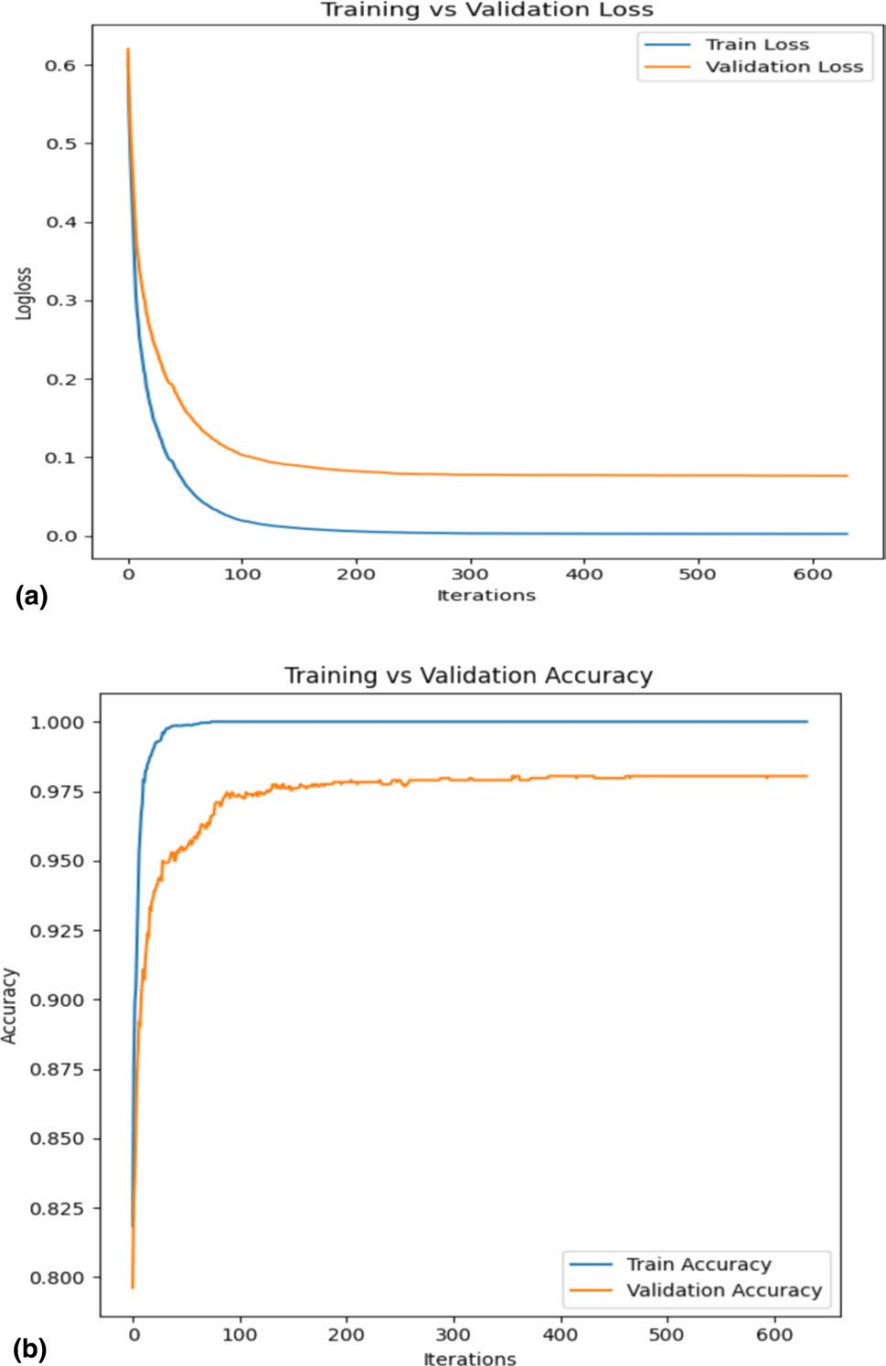
Training loss and validation loss are represented by (**a**), versus Training accuracy and validation accuracy are represented by (**b**).

The confusion matrix provides a useful visual depiction of the model’s performance and can help guide future improvements. Our study used a confusion matrix to evaluate the performance of the proposed model. Figure 7 shows the confusion matrix for the proposed model, which accurately predicted 1346 out of 1379 occurrences, with false positives being 20 and false negatives being 7 occurrences. In Fig. 8, accuracy and loss of the validation dataset are shown. Training loss and validation loss in Fig. 9a versus the training accuracy and validation accuracy in Fig. 9b highlight that the proposed hybrid model is well fitted.

### Explanation of the prediction of the proposed model

**Fig. 10:**
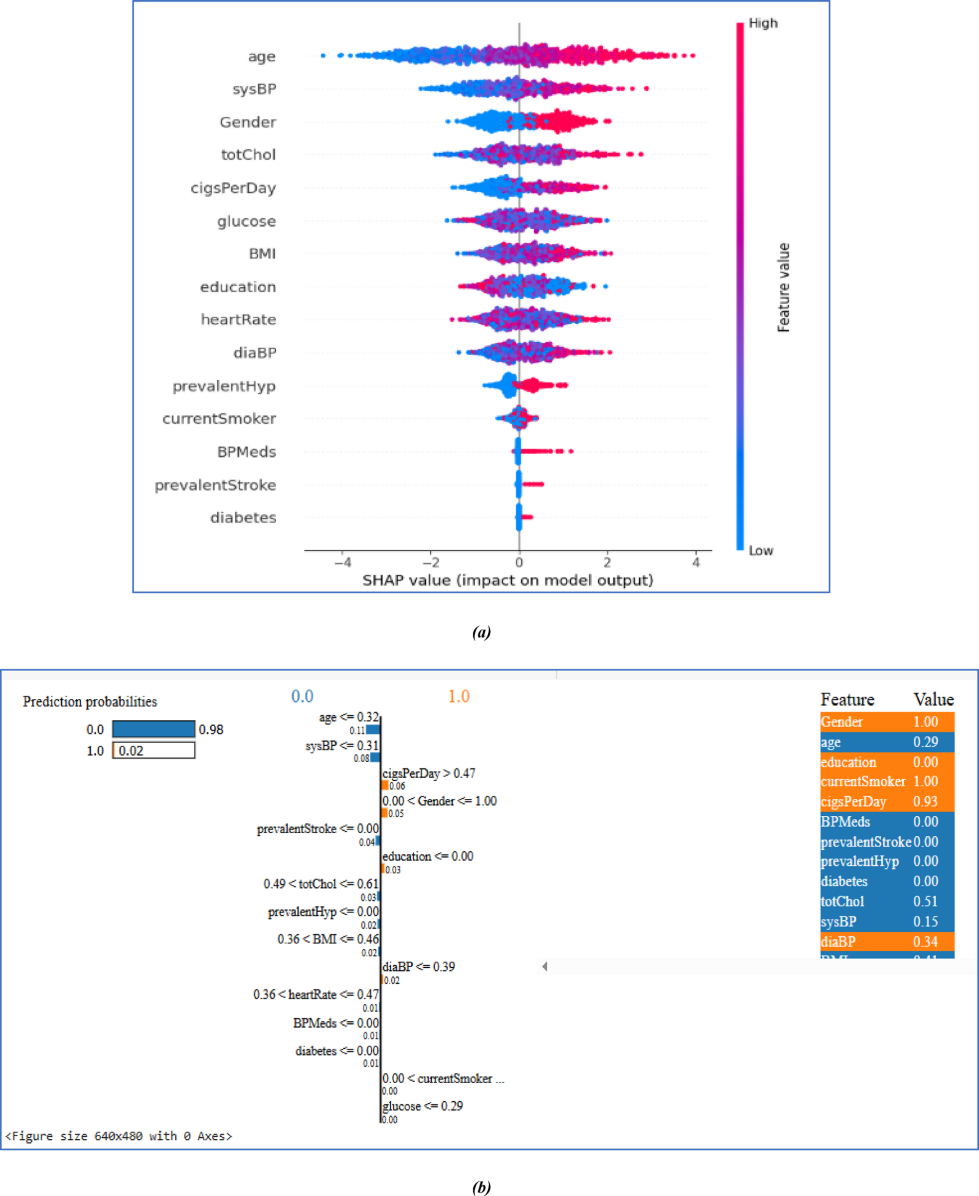
(**a**,** b**) Visualization of Significant Features with SHAP and LIME value, respectively.

Figure 10a SHAP summary graphic shows how important features are and how they affect model predictions globally. The most influential factors are **‘age’**, **‘sysBP’**, and **‘Gender’**, with **‘diabetes’** and **‘prevalent stroke’** contributing the least. Each dot represents a data point, with positive SHAP values improving predictions and negative ones lowering them. The colour gradient reflects feature values, with red representing greater values and blue representing lower values. Higher **‘glucose’** and **‘BMI’** levels are associated with a higher risk. Lifestyle variables such as **‘Current smoker’**, **‘BMI’**, and **‘PrevalentHyp’** considerably influence forecasts, as do categorical traits such as **‘education’**. This approach identifies **age, systolic blood pressure, and cigarettes per day** status as prominent risk variables, increasing model interpretability and revealing significant cardiovascular disease predictors. In Fig. 10b, this graphic explains the proposed model’s prediction using LIME as a local interpretability approach. The left side shows the prediction probabilities, with the model favouring class 0 (98%) over class 1 (2%). It is very clearly shows that the particular patient has no chances to having CVD in upcoming ten years because of those feature **‘age’,** ‘**sysBP’, ‘Prevalent Stroke ‘, ‘totChol’,** **‘prevalentHyp’, ‘BMI’, ‘heartRate’, ‘BP Meds’, ‘diabetes’,** leading it 0.98 of probability to No CVD and rest of Features like **‘cigsPerDay’, ‘ Gender ‘, ‘Education’, ‘diaBP’, ‘Current smoker’, ‘Glucose’,** are causes of 0.02 of probability of CVD. The middle part depicts the decision route, emphasizing the critical circumstances that drove the forecast. The right-side lists contributions, with Gender (1.00), Current Smoker (1.00), and CigsPerDay (0.93) having the most influence. Orange-shaded features pushed the prediction towards class 1, but blue-shaded features contributed to class 0, demonstrating a more significant effect in predicting a lower-risk result. This image aids in understanding how various characteristics influence the model’s decision-making.

### Comparison with existing works on the same dataset

**Fig. 11:**
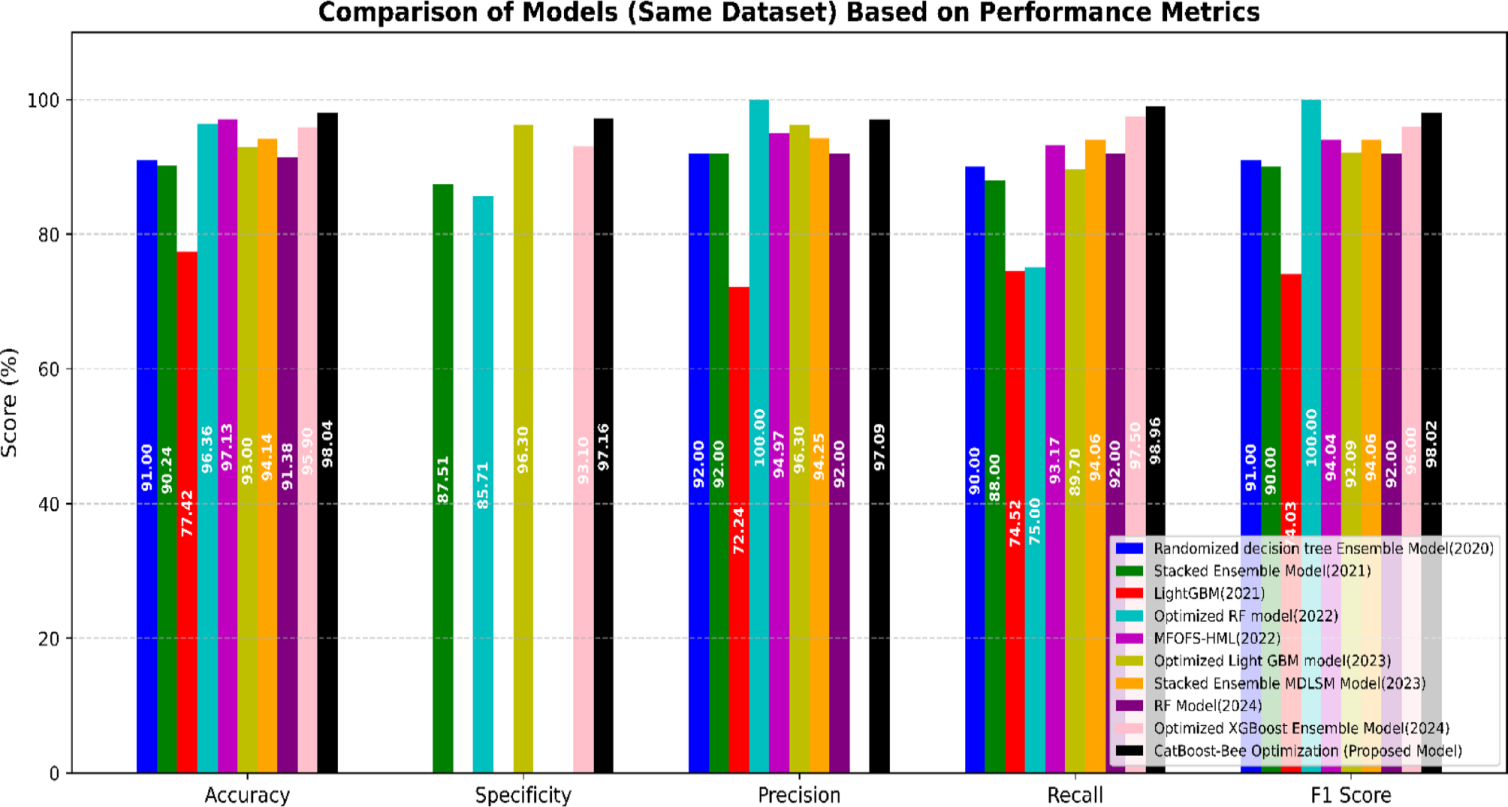
Comparison of evaluation matrices of the proposed model with the existing approaches on the same dataset.

The comparison of models based on performance metrics in Table 7 and the bar chart in Fig. 11 shows that the **CatBoost-Bee Optimization (Proposed Model)** outperforms all other approaches evaluated on the same dataset. While earlier models, such as the **Randomized Decision Tree Ensemble Model (2020)** and the **Stacked Ensemble Model (2021)** delivered moderate results, and the **LightGBM (2021)** model showed the weakest performance overall, more recent models like the **Optimized RF Model (2022)** and **MOFOS-HML (2022)** demonstrated improvements but still lacked consistency across all metrics. The **Optimized Light GBM Model (2023)** and **Stacked Ensemble MDLSM Model (2023)** achieved balanced scores but did not lead in any metric. Even advanced models from 2024, such as the **RF Model** and the **Optimized XGBoost Ensemble Model**, while competitive and strong in specific areas like recall and precision, fell slightly short in overall performance. In contrast, the **CatBoost-Bee Optimization (Proposed Model)** achieved the highest scores across all five metrics: **Accuracy (98.04%)**, **Specificity (97.16%)**, **Precision (97.09%)**, **Recall (98.96%)**, and **F1 Score (98.02%)**. This consistent dominance in every category highlights its exceptional robustness, precision, and balance, clearly establishing it as the most effective and reliable model among all those compared.

**Table 7:** Comparison of our proposed hybrid model with state-of-the-art methods on the same dataset (**MFOFS-HML**: A Mayfly Optimization-based Feature Selection method with a hybrid Machine Learning model, consisting of a **Convolutional Neural Network (CNN)** and a **Hopfield Neural Network (HNN)**).

Year	Techniques	Accuracy (%)	Specificity (%)	Precision (%)	Recall (%)	F1-score (%)	XAI method
2020^47^	Randomized Decision Tree Ensemble Model	91	X	92	90	91	X
2021^48^	Stacked Ensemble Model	90.24	87.51	92	88	90	X
2021^49^	LightGBM	77.42	X	72.24	74.52	74.03	X
2022^50^	Optimized RF model	96.36	85.71	100	75	100	X
2022^51^	MFOFS-HML Model	97.13	X	94.97	93.17	94.04	X
2023^52^	Optimized Light GBM Model	93.00	96.30	96.30	89. 70	92.09	X
2023^53^	Stacked Ensemble MDLSM Model	94.14	X	94.25	94.06	94.06	X
2024^54^	RF Model	91.38	X	92	92	92	SHAP
2024^55^	Optimized XGBoost Ensemble Model	95.90	93.10	X	97.50	96.00	SHAP
	CatBoost-Bees Optimization (Proposed Model)	98.04	97.16	97.09	98.96	98.02	LIME, SHAP

## Conclusions

CVD is a disease that affects the blood vessels in the heart, often leading to strokes, heart attacks, and even heart failure. The disease has a significant impact on global health, being one of the leading causes of death across the world. The present study emphasizes the detection of CVD using hybrid machine learning approaches to enable proactive treatment and efficient decision-making. The dataset used in the study is the Framingham Heart Study dataset collected from the publicly available Kaggle repository. The data is prepared at the outset by eliminating duplicates and imputing missing values. Then the data is pre-processed by removing outliers, balancing the dataset using the RO technique, followed by Pearson correlation to understand dependability, and Min–Max scaling for data normalization. The pre-processed data is fed into a hybrid ML framework wherein the CatBoost classifier algorithm and hyperparameter optimization using the Bees algorithm are performed. The model’s results are evaluated using Accuracy, Precision, Recall, F1-Score, Specificity, ROC, and PRC metrics. The proposed model achieved an impressive accuracy of 98.04%, yielding a superior outcome compared to the contemporary approaches. Further, the model’s outcome was compared with state-of-the-art methods implemented on the same datasets, validating its superiority. To achieve interpretability, XAI techniques, namely LIME and SHAP, were implemented to analyse the significance of the attributes contributing to the risks of CVD occurrences. This gave better insights, enabling health care professionals to make informed decisions, leading to higher success rates in treating the disease. Although the proposed model yielded promising results and enhanced visualization and interpretability, the study did not include real-time data. As a future research direction, real-time data could be used to get an improved perspective, enabling the model to be used confidently in real-time patient treatment. The proposed system is technically, operationally, and economically feasible. It presents a robust, accurate, and interpretable solution for early CVD prediction. Its integration with XAI makes it suitable for clinical environments where explainability is essential. Future developments should focus on real-world validation, external datasets, and potential clinical integration.

### Limitations

The study’s CatBoost-BEEs model shows high accuracy. Still, its reliance on a single dataset, lack of real-time validation, missing clinical variables, and use of a less-common optimization method limit its generalizability and clinical applicability. It also focuses only on binary classification, not disease severity.

## Data Availability

In this proposed work, the data used for analysis are taken from the data source Kaggle repository, [https://www.kaggle.com/datasets/aasheesh200/framingham-heart-study-dataset]. Accessed in January 2024.
